# Assessing spontaneous sensory neuron activity using in vivo calcium imaging

**DOI:** 10.1097/j.pain.0000000000003116

**Published:** 2023-12-15

**Authors:** Sonia Ingram, Kim I. Chisholm, Feng Wang, Yves De Koninck, Franziska Denk, George L. Goodwin

**Affiliations:** a Sonia Ingram, Data Scientist, Contract Researcher for King's College London, London, United Kingdom; b Pain Centre Versus Arthritis, School of Life Sciences, University of Nottingham, Nottingham, United Kingdom; c CERVO Brain Research Centre, Québec Mental Health Institute, Quebec City, QC, Canada; d Faculty of Dentistry, Laval University, Quebec, Canada; e Wolfson Centre for Age-Related Diseases, King's College London, London, United Kingdom

**Keywords:** Spontaneous activity, In vivo calcium imaging, Peripheral pain mechanisms

## Abstract

Heightened spontaneous activity in sensory neurons is often reported in individuals living with chronic pain. It is possible to study this activity in rodents using electrophysiology, but these experiments require great skill and can be prone to bias. Here, we have examined whether in vivo calcium imaging with GCaMP6s can be used as an alternative approach. We show that spontaneously active calcium transients can be visualised in the fourth lumbar dorsal root ganglion (L4 DRG) through *in vivo* imaging in a mouse model of inflammatory pain. Application of lidocaine to the nerve, between the inflamed site and the DRG, silenced spontaneous firing and revealed the true baseline level of calcium for spontaneously active neurons. We used these data to train a machine learning algorithm to predict when a neuron is spontaneously active. We show that our algorithm is accurate in 2 different models of pain: intraplantar complete Freund adjuvant and antigen-induced arthritis, with accuracies of 90.0% ±1.2 and 85.9% ±2.1, respectively, assessed against visual inspection by an experienced observer. The algorithm can also detect neuronal activity in imaging experiments generated in a different laboratory using a different microscope configuration (accuracy = 94.0% ±2.2). We conclude that in vivo calcium imaging can be used to assess spontaneous activity in sensory neurons and provide a Google Colaboratory Notebook to allow anyone easy access to our novel analysis tool, for the assessment of spontaneous neuronal activity in their own imaging setups.

## Abstract 2

Supplemental Digital Content is Available in the Text.

In vivo calcium imaging is a suitable technique for studying spontaneous sensory neuron activity in models of pain.

## 1. Introduction

Spontaneous activity in the nervous system is defined as the generation of neural activity without any appreciable external task or stimulus. In peripheral sensory neurons, this type of activity is rarely found in healthy individuals.^19,22^ Instead, it is prevalent in nociceptors of many people with chronic neuropathic^14,21,27^ and musculoskeletal^2,28^ conditions, and it is associated with spontaneous pain.^20^ Increased spontaneous sensory neuron activity has also been reported in rodent models of chronic pain.^7,24,27,29^ Unsurprisingly, given this evidence, pharmaceutical companies have focused efforts on developing analgesics that can normalise this hyperactivity, by targeting ion channels and receptors located in sensory neurons, in the hope of blocking the generation of unwanted pain.^10,13^ Yet, this strategy has so far failed to produce effective analgesics. One reason why it might be difficult to make progress in this area is that we lack basic mechanistic knowledge.

Traditionally, spontaneous activity has been studied using in vivo electrophysiology, which typically involves one-by-one recordings from individual sensory nerve filaments using a technically challenging teased-fibre setup. We are ourselves one of the few groups that are proficient in this technique^9,11^ and are therefore familiar with its laborious nature. Nociceptive C-fibres are particularly difficult to record from, due to their biophysical properties. Specifically, their small size and large resistance cause their extracellular spike amplitudes to be very small, and this requires the nerve to be teased into extremely fine filaments to achieve a sufficiently high signal-to-noise ratio. Such technical challenges mean that these studies are lengthy and low throughput, usually reporting only on around 10 to 15 fibres per animal. They are also prone to bias. For example, the smallest C-fibres are likely to be undersampled because they are the first to disintegrate when teased. Moreover, receptive field searching for nociceptors can cause neuronal sensitization, and an increase in the proportion of spontaneously active fibres can be found.^1^

In vivo calcium imaging is a relatively new method for studying sensory neuronal function, which can be used to study hundreds of cells simultaneously.^5^ Given the difficulties associated with studying spontaneous activity through electrophysiology, we set out to determine whether in vivo imaging could be used as an alternative, to improve our understanding of this phenomenon. For this, we used an inflammatory pain model to observe spontaneously active calcium transients in GCaMP6s mice. To differentiate spontaneous activity from unrelated fluctuations in fluorescence, we used a local anaesthetic to block incoming electrical activity from the inflamed site. These data were then assessed by an experienced observer, to identify which neurons were spontaneously active. Using these “ground truth” data, we trained an algorithm to detect spontaneously active neurons (without the need for lidocaine application). We confirmed the robustness of the algorithm by testing it on independent data in 2 different pain models and on data generated using a different microscope setup in an independent laboratory. Our results suggest that in vivo imaging with GCaMP6s is suitable for the large-scale assessment of spontaneous activity in sensory neurons.

## 2. Materials and methods

### 2.1. Animals

Animal experiments conducted in the United Kingdom: Adult C57BL/6J male and female mice (n = 32; Charles River, United Kingdom) weighing 24 to 30 g were used in this study. Mice were housed on a 12/12 hours light/dark cycle with a maximum of 5 mice per cage, with food and water available ad libitum. All experiments were performed in accordance with the United Kingdom Home Office Animals (Scientific Procedures) Act (1986).

Animal experiments were conducted in Canada: All experiments were performed in accordance with regulations of the Canadian Council and Animal Care. Homozygous MrgprD-Cre mice (B6.129S1(Cg)-*Mrgprd*^*tm1.1(cre)And*^/Mmnc, MMRRC, 036118-UNC) were crossed with C57BL/6J mice (JAX, 000664) to have MrgprD-Cre heterozygous mice. A total of 6 adult mice were used in 2-photon recording experiments.

### 2.2. Administration of GCaMPs

We used the genetically encoded calcium indicator GCaMP6s for imaging sensory neuron activity.^4^ GCaMP6s was delivered to sensory neurons through an adenoassociated viral vector of serotype 9 (AAV9), which was administered to mouse pups at P2-P7 as previously described.^34^ Briefly, groups of 3 to 4 mice were separated from their mother. Five microliter of AAV9.CAG.GCaMP6s.WPRE.SV40 or AAV9.syn.GCaMP8s-WPRE virus (Addgene, Watertown, MA) was injected subcutaneously in the nape of the neck, using a 10-µL Hamilton syringe with a 30-G needle. Pups were placed back into their home cage after the injection was complete. Mice were separated from their mother after weaning and then used for in vivo imaging from 10 weeks after the injection. These mice were used for 1-photon imaging experiments.

For validation of our algorithm, preexisting data from another laboratory was used. These data had been generated for the purposes of an entirely different study. In that study, nonpeptidergic nociceptors were specifically targeted for imaging, using intraplantar injections of 10 µL of AAV9.CAG.Flex.GCaMP6s.WPRE.SV40 (Addgene, 100842-AAV9) into newborn MrgprD-Cre+ mice. Imaging was performed using a 2-photon microscopy.

### 2.3. Complete Freund Adjuvant pain model

To induce acute inflammation and pain, 20-µL complete Freund adjuvant (CFA; 1 mg/mL; #SLBZ9884, Sigma, Gillingham, United Kingdom) was injected intraplantar into the left hind paw using a 30-G insulin syringe.

### 2.4. Antigen-induced Arthritis model

Mice were immunised using an emulsion of CFA (3.3 mg/mL; #1216771 Scientific Laboratory Supplies, Nottingham, United Kingdom) and mBSA (40 mg/mL; #SLBN1991V, Sigma), as described previously.^35^ Briefly, mice were anesthetised using 2% isoflurane, and 100 µL of the emulsion was subcutaneously injected at the base of the tail and in the right flank (50 µL each). Mice were then allowed to recover and were returned to their home cages. Seven days after immunisation, mice were anaesthetised with isoflurane, and 2.5 to 5 µL of mBSA (5µl n = 4, 2.5µl n = 2; 200µg/injection) was injected into the left knee joint using a Hamilton syringe with a 30-G needle.

### 2.5. In vivo imaging of sensory neuron activity using GCaMP6s

#### 2.5.1. 1-photon imaging

Mice were anesthetized using a combination of drugs: 1 to 1.25 g/kg 12.5% wt/vol urethane administered intraperitoneally and 0.5% to 1.5% isoflurane delivered through a nose cone. Body temperature was maintained close to 37°C using a homeothermic heating mat with a rectal probe (FHC, Bowdoin, ME). An incision was made in the skin on the back, and the muscle overlying the L3, L4, and L5 DRG was removed. Using fine-tipped rongeurs, the bone surrounding the L4 DRG was carefully removed in a caudal–rostral direction. Bleeding was prevented using gelfoam (Spongostan; Ferrosan, Denmark). The DRG was washed and kept moist using 0.9% sterile saline. The position of the mouse varied between prone and lateral recumbent to orient the DRG in a more horizontal plane. The exposure was then stabilized at the neighbouring vertebrae using spinal clamps (Precision Systems and Instrumentation, Fairfax Station, VA) attached to a custom-made imaging stage. We strongly advise exercising particular care during the stabilisation step because this has been found to be one of most prominent ways of introducing excess noise into the signal. To ensure the accuracy of our automatic detection of spontaneous activity, we suggest that no movement of the DRG should be visible through a standard dissection microscope. Finally, the DRG was covered with silicone elastomer (World Precision Instruments, Ltd, Hertfordshire, United Kingdom) to maintain a physiological environment. Before imaging, we administered a subcutaneous injection of 0.25 mL of 0.9% sterile saline to keep the mouse hydrated. It was then placed under an Eclipse Ni-E FN upright confocal/multiphoton microscope (Nikon, Surbiton, United Kingdom), and the microscope stage was diagonally orientated to optimise focus on the L4 DRG. The ambient temperature during imaging was kept at 32°C throughout. All images were acquired using a 10× dry objective. A 488-nm Argon ion laser line was used to excite GCaMP6s, and the signal was collected at 500 to 550 nm. Time-lapse recordings were taken with an in-plane resolution of 512 × 512 pixels and a partially (∼3/4) open pinhole for confocal image acquisition. All recordings were acquired at 3.65 Hz. Mice were culled with an overdose of sodium pentobarbital at the end of each experiment.

#### 2.5.2. 2-photon imaging

Imaging was performed as previously described.^34^ Briefly, adult MrgprD-Cre mice (after viral injection) were deeply anesthetized intraperitoneally with 100 mg/kg of ketamine, 15 mg/kg of xylazine, and 2.5 mg/kg of acepromazine (A7111, Sigma-Aldrich, Burlington, MA).^6,32^ Laminectomy was performed to expose L4 DRG, and the spinal columns flanking the laminectomy exposure were clamped with 2 clamps of a home-made spinal stabilization device to fix the animals. Three percent agar solution was used to make a pool for holding Ringer solution (in mM: 126 NaCl, 2.5 KCl, 2 CaCl2, 2 MgCl2, 10 D-Glucose, 10 HEPES, pH = 7.0). Dextran Texas Red (70 kDa, Neutral, D1830, Invitrogen; 1% in saline) was injected intravenously to label the blood vessels, which provide structural landmarks for image registration. The animals were heated with a heating pad during the surgery and imaging to keep the body temperature at 37°C. Warmed Ringer solution was dropped on the exposed spinal cord and DRGs to keep the moisture, and repeatedly changed during the imaging experiment. Animals with the whole spinal stabilization device were fixed under a homemade video-rate 2-photon microscope. A tunable InSight X3 femtosecond laser (Spectra-Physics, Milpitas, CA) was set to 940 nm for GCaMP6s and Texas Red imaging. Images were acquired at 32 Hz with an Olympus water-immersion 40x objective at a resolution of 0.375 µm/pixel.

### 2.6. Calcium imaging data processing

#### 2.6.1. 1-photon recordings

Time-lapse recordings were concatenated and scaled to 8-bit in Fiji/ImageJ, Version 1.53. The image analysis pipeline Suite2P (https://github.com/MouseLand/suite2p; v 0.9.2)^23^ was used for motion correction, automatic region of interest (ROI) detection, and signal extraction. Further analysis was undertaken with a combination of Microsoft Office Excel 2013, Matlab (2018a), and RStudio (Version 4.02). A region of background was selected, and its signal subtracted from each ROI. To generate normalised ΔF/F0 data, the ROMANO toolbox ProcessFluorescentTraces() function was used.^25^ This function uses the calculation: ΔF/F0 = (Ft − F0)/F0, where Ft is the fluorescence at time t, and F0 is the fluorescence average over a baseline period. ΔF/F0 is expressed as a percentage.

#### 2.6.2. 2-photon recordings

The recorded images were processed and analysed as previously reported.^34^ Briefly, the RAW image sequences were first converted into TIFF format in ImageJ. Then rigid body translation alignment based on the 2-dimensional cross-correlation was performed with a custom-built MATLAB (MathWorks, Natick, MA) function to correct for movement (image registration). A rectangular region of interest (ROI) in a region without any visible neuron was drawn as background ROI. The average pixel value inside the background ROI for each frame was subtracted from every pixel in the corresponding frame to remove excess noise. Then, small rectangular ROIs were placed manually in the cytoplasm of individual visible neurons. The average fluorescence intensity of a given ROI, Ft, was measured by averaging pixel values inside the ROI. Calcium traces were calculated as, ΔF/F0 = (Ft − F0)/F0, where F0 is the fluorescence value at baseline, which was measured as the average of the first 2 seconds of Ft. To avoid aberrant amplification because of small F0 values in some neurons (eg, low basal fluorescence), when it was <1, F0 in the denominator, but not in the numerator, was set to 1. The resulting Ca2+ time series extracted from the image sequences were synchronized with thermal or mechanical stimulus data series. Processing was performed using custom functions written in MATLAB.

An integrated interface within a custom tool written in Spike2 (CED) was used to automatically detect and measure positive responses to stimuli. Raw Ca2+ traces were first smoothed with a 1-second temporal window. Baseline fluorescence (Fb) was selected from a period between 1 second after the beginning of the recording and 1 second before the stimulus onset (usually 3-5 seconds in duration). Then, Fb and Fb-max were calculated as average and maximum ΔF/F0 values during baseline, respectively. A response was considered positive when the peak of the Ca2+ trace during stimulation was above Fb + (Fb-max-Fb) × x, where x was a value between 2 and 3, depending on baseline stability, to provide the most reliable detection. Given the relatively slow decay of GCaMP6s, responses with very brief duration (<0.5 seconds) were excluded. All traces were also visually inspected to ensure that no false positives were included and no false negatives missed.

Before running these data through the spontaneous activity detection algorithm, it was downsampled from 32 Hz to 3.65 Hz (acquisition speed using for training algorithm) using a linear interpolation function in python.

### 2.7. Mechanical and heat stimulation of the peripheral terminals

Neurons in the leg and foot were stimulated through mechanical stimulation. Specifically, mechanically sensitive afferents were identified by both pinching, using a pair of serrated forceps, and brushing with a paintbrush and by moving the foot.

A feedback-controlled Peltier device and a 1 cm × 1 cm thermal probe (TSA-II-NeuroSensory Analyzer, Medoc, Ramat Yishay, Israel) were used to deliver noxious heat stimulation in a fast ramp and hold mode to the plantar side of the hind paw. The speed of temperature increase was set at 8°C/second, and temperature decrease was set at 4°C/second. The baseline temperature was set at 25°C, and the duration of the steady-state phase of the 50°C stimulation was 5 seconds.

### 2.8. Electrical stimulation of the sciatic nerve

In some experiments, the sciatic nerve was dissected at the level of the midthigh and freed of any surrounding connective tissue. A custom-made electrical stimulation cuff made of steel wire was fitted under the nerve, and square wave pulses of 1-millisecond width and 5 mA amplitude (suprathreshold) were applied at 0.2 Hz for 5 minutes.

### 2.9. In vivo lidocaine application

Lidocaine hydrochloride (Sigma; dissolved in 0.9% wt/vol sodium chloride to a concentration of 74 mM, 2% wt/vol) was used to block any voltage-gated sodium channel–dependent spontaneous activity, thereby revealing the true baseline level of calcium in these neurons. Lidocaine injection directly into the native sciatic notch during the recording was challenging. Therefore, to increase the accuracy of our injections onto the sciatic nerve, we “marked up” the position of the notch by making a small incision in the skin and surrounding muscle at the level of the midthigh before recording. For mice that were used for spontaneous activity testing, no incisions were made in the leg before recording baseline activity. To accurately inject onto the nerve, the mouse was removed from the microscope stage, and the sciatic nerve was exposed at the level of the midthigh. The mouse was then returned to the stage, and the imaging plane was carefully realigned. After 2 minutes of recording, lidocaine was applied to the nerve.

### 2.10. Machine learning algorithm

The machine learning algorithms used in this study are available as part of the Alan Turing institute Sktime project (https://www.sktime.org/en/stable/).^15,16^ Preliminary investigations demonstrated that 2 interval-based algorithms were most effective with this data set: time series forest classifier (TSF) and random interval spectral ensemble (RISE). Both algorithms are forest based, allocating intervals of the input time series to individual tree classifiers. The outputs of individual trees are fed forward toward an ensemble, which calculates the final model prediction. Time series forest divides the entire time series into *n* distinct intervals, calculates the mean, standard deviation, and slope of each interval, and uses these 3 features for its tree classifiers. By contrast, RISE converts random time intervals to spectral coefficients, which are used as features for its tree classifiers.

Model training was performed using an 80:20 ratio train test split in concert with K-fold cross validation (k = 5). Model training and serving are available through a Google Collab notebook: https://github.com/sonialouise/ts_class/blob/main/notebooks/SpontaneousActivity.ipynb and all code is available through Github: https://github.com/sonialouise/ts_class.

### 2.11. Algorithm training and testing

**Figure 1.**
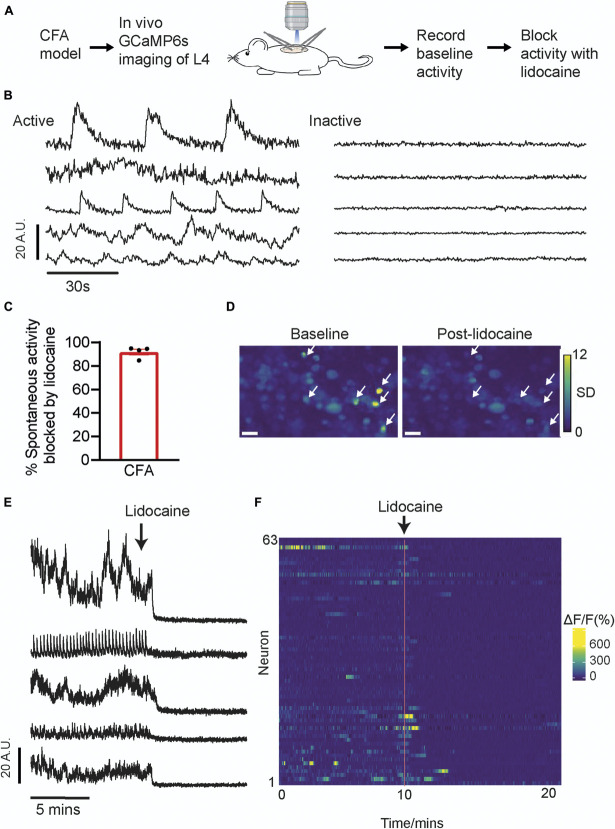
Examination of inflammation-induced spontaneously active calcium transients. Experimental schematic is shown in (A). Example fluorescent traces of spontaneously active and inactive neurons are shown in (B). The bar graph shows the proportion of CFA-induced spontaneously active neurons that were blocked by lidocaine application to the sciatic nerve (C). Example images before and after lidocaine application (D). Images were generated using a z-projection of the standard deviation of 500 frames at baseline (left panel) and postlidocaine application (right panel). Arrows indicate examples of neurons that were spontaneously active at baseline and silent post-lidocaine. Further examples of spontaneously active neurons are shown in the fluorescent traces (E) and in the heat map of ΔF/F0 normalised data (F). Lidocaine application to the sciatic nerve (timing indicated by arrows in E and F) in between the inflamed site and the DRG recording site confirmed that spontaneous activity was peripherally generated and revealed the true baseline of these neurons. Scale bar = 50 µm. See also Supplementary Video 1. CFA, complete Freund adjuvant; DRG, dorsal root ganglion.

The algorithms were trained on neurons that were deemed “active” or “inactive,” and this was determined as follows. Spontaneous activity was induced using the CFA model, with mice imaged on day 1 or day 2 postinjection. Spontaneously active neurons were differentiated from those which were silent using lidocaine, which, when applied to the nerve in between the inflamed site and the DRG, blocks any incoming action potentials that are being generated in the inflamed paw (Fig. 1A). Block by lidocaine was determined by examining calcium traces by eye (assessed by an experienced observer). Fluorescent traces were taken from spontaneously active neurons during the first (prelidocaine) and last (5 minutes postlidocaine) 1100 frames (∼5 minutes), to generate “active” and “inactive” training data sets, respectively. Traces in which activity before and after lidocaine was ambiguously altered were excluded from training data sets, ie, they may have been active spontaneously, but their activity might have been generated at a site remote from the inflamed paw. The number of frames used for testing must not exceed the number of frames used for training. We have used 301 seconds (∼5 minutes) data segments for training, and therefore, this is the maximum duration that can be used for testing with our current training data sets.

Electrophysiological experiments indicate that most neurons firing spontaneously in the CFA pain model are nociceptors.^8,37^ To ensure that the algorithm was trained on baseline calcium traces in other neuron types, ie, nonnociceptors, the postlidocaine data (>5 minutes after application) from neurons in the DRG that were not spontaneously active was included in the “inactive” neuron training set. It was assumed that these neurons would be silent because, under normal conditions, activity in sensory neuron is typically generated at the peripheral terminals (which were blocked), and indeed, this was true for most experiments (confirmed by visually inspecting traces).

We compared the consequences of training the algorithm with 2 different types of calcium imaging data: raw fluorescent calcium traces or normalized traces using ΔF/F0. Each version was then tested for prediction accuracy using independent data sets. Unless otherwise stated, sensitivity, specificity, and accuracy were calculated as follows:

$$Accuracy=\frac{TP+TN}{TP+TN+FP+FN}$$

$$Sensitivity=\frac{TP}{TP+FN}$$

$$Specificity=\frac{TN}{TN+FP}.$$

True positive (TP) = the number of neurons with spontaneous activity (as determined by lidocaine). False positive (FP) = the number of neurons incorrectly identified as active. True negative (TN) = the number of neurons without spontaneous activity (as determined by lidocaine). False negative (FN) = the number of neurons incorrectly identified as inactive.

The data set acquired using 2P microscopy did not involve the application of lidocaine, nor were they conducted in a disease context in which one would expect spontaneous activity. However, they were designed to induce neuronal firing in response to heat and mechanical stimulation. Therefore, we conducted a blinded trial to assess our algorithm's ability to detect evoked activity in these 2-photon recordings. Active neurons were determined using a custom-written tool in Spike2, which can accurately detect evoked but not spontaneous activity, and then, the results were compared with those generated using the machine learning algorithm.

### 2.12. Quantification and statistical analysis

Graphing and statistical analysis was undertaken with a combination of Microsoft Office Excel 2013, R Studio (Version 4.02) and GraphPad Prism (Version 8). Details of statistical tests and sample sizes are recorded in the appropriate figure legends. All data plotted represent mean ± SEM. Unpaired Student *t* tests were used to compare the proportion of spontaneously active neurons between treatment and control groups.

## 3. Results

### 3.1. Assessment of spontaneous activity in an inflammatory pain model

We chose the CFA model to study spontaneous activity because a high proportion of sensory neurons are reported spontaneously active at early time points in electrophysiology studies.^8,37^ As expected, GCaMP6s mice that were imaged using a 1-photon confocal microscope 1 to 2 days post-CFA injection showed increased spontaneous calcium transients that resembled spontaneous activity (Fig. 1B—left panel). Application of lidocaine in between the inflamed hind paw and the DRG recording site blocked the majority of spontaneous calcium transients that were observed in CFA animals (91.9% ±2.4, n = 4 mice; Fig. 1C). This confirmed that the activity was generated by action potentials, ie, not caused by movement artefacts, and that it was generated in the periphery (Figs. 1D–F, Supplementary Video 1).

Spontaneous activity recorded in the DRG may itself originate in the soma of primary afferents, rather than in the axon.^33^ Therefore, it should be noted that we were likely unable to inhibit all spontaneous activity through the application of lidocaine onto the nerve. Lidocaine application to the DRG was not possible due to our recording set up (see methods), and as a result, we were not able to quantify the total number of spontaneously active neurons in the DRG. Indeed, the skill required to apply lidocaine directly onto the nerve while visualising the DRG or, more difficult still, applying lidocaine directly to the DRG (to remove remaining ectopic spontaneous activity), presents a significant barrier to the study of spontaneous activity in the peripheral nervous system. In addition, the added step of lidocaine application to any parts of the peripheral nervous system often does not fit easily within a given experimental paradigm. Therefore, we set out to develop an algorithm that could be used on any baseline recordings to accurately predict when a neuron is firing, including on data already recorded for other purposes.

### 3.2. Development of an algorithm to detect spontaneous activity in in vivo calcium imaging recordings

**Figure 2.**
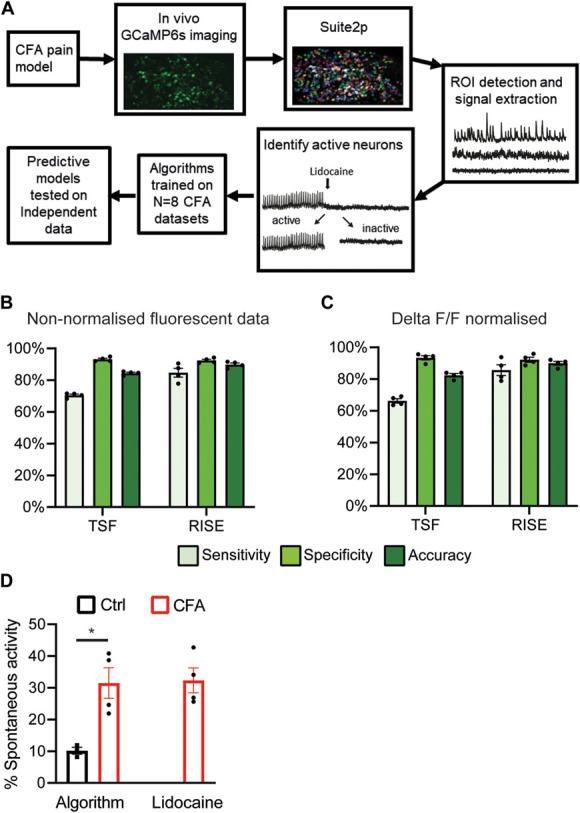
Training machine learning algorithms to detect spontaneous activity in GCaMP6s DRG recordings. Schematic shows the workflow of how CFA-induced spontaneously active neurons were identified for algorithm training (A). The algorithms were tested on n = 4 independent CFA experiments, after having been trained on either non-normalised (B) or ΔF/F0 normalised (C) data. Sensitivity = the ability of the algorithm to correctly identify when a neuron is spontaneously active. Specificity = the ability of the algorithm to correctly identify when a neuron is not active. Accuracy = the ability of the algorithm to differentiate spontaneously active from inactive neurons. Lidocaine was used to establish ground truth data, ie, determine whether a neuron was spontaneously active or not. The proportion of neurons in the L4 DRG that were predicted to have spontaneous activity in control and CFA animals on day 1 is shown in (D). Left side—algorithm prediction, right side—conventional quantification by experimenter. Note that our data sets only include information on spontaneously active neurons blocked by lidocaine application to the nerve, ie, not those that might have had activity originating in the cell soma. n = 3-4/group Bar graphs represent mean ± SEM. Each dot represents data from one animal. * p<0.05. CFA, complete Freund adjuvant; DRG, dorsal root ganglion; RISE, random interval spectral ensemble; TSF, time series forest classifier.

To assemble a “ground truth” data set of spontaneously active neurons, we performed imaging experiments on n = 8 CFA mice. In each experiment, lidocaine nerve block was used to distinguish spontaneous activity from baseline noise (Fig. 2A). We believed that perhaps a measure of variance, such as the standard deviation, could predict spontaneous activity. However, we found that the standard deviation of spontaneously active and inactive segments overlapped more than previously anticipated: 77.9% of neurons with no activity and 23.1% of those with activity had standard deviations of below 15% (ΔF/F0; Supplementary Fig. 1A, available at http://links.lww.com/PAIN/B956). We applied basic formulas using the standard deviation to distinguish activity from noise and could not identify an acceptable compromise between sensitivity and specificity, ie, increasing the ability of the algorithm to detect true spontaneously active neurons would lead to a very high rate of false positives and vice versa. A threshold for spontaneous activity of >2.5 × the standard deviation on more than 30 occasions in 5 minutes performed the best on our ground truth data, but prediction accuracies were not reproducible when testing on independent CFA experiments (accuracy = 78.0% ±0.6; Supplementary Fig. 1B, available at http://links.lww.com/PAIN/B956). Therefore, we decided to investigate whether supervised machine learning algorithms specialised for time series data could provide better predictive accuracies. We trained 2 different algorithms, using the visual lidocaine block as “ground truth” data, on both ΔF/F0 normalised and non-normalised raw fluorescent data (1308 neurons, 272 active, and 1036 inactive from n = 8 CFA mice). The 2 algorithms that were tested were the TSF and RISE. Both algorithms are forest based and were selected because they performed best on our data sets in preliminary testing.

Upon testing on n = 4 independent CFA data sets (225-316 neurons/experiment), we found that the normalisation method (raw fluorescence vs deltaF/F0) had little impact on the predictive accuracy of our algorithms (Figs. 2B). However, for both normalisation methods, the RISE algorithm predicted spontaneous activity more accurately than the TSF algorithm (TSF accuracy = 84.4% ±0.6, RISE accuracy 87.2% = ±1.0; TSF accuracy = 82.4% ±1.1, RISE accuracy = 90.0% ±1.2, respectively, for non-normalised and normalised data; Fig. 1C). Therefore, going forward, we opted to use the RISE algorithm trained on ΔF/F0 normalised data because it performed best in these initial tests and is likely more generalisable (ΔF/F0 being the most dominant processing step in the field).

Reducing the number of frames the algorithm was trained and tested on from 1100 to 550 or 200 did not markedly reduce the algorithms prediction accuracy performance (RISE accuracies = 88.9% ±1.7, 88.3% ±1.7 for 550 and 200 frame training respectively; n = 4 mice; Supplementary Fig. 2A & B, available at http://links.lww.com/PAIN/B956). Reducing the sampling rate to 3 Hz using a linear interpolation function in Python caused a small reduction in accuracy (TSF accuracy = 81.8% ±0.8, RISE accuracy 85.6% ±0.7; Supplementary Fig. 3, available at http://links.lww.com/PAIN/B956). By contrast, downsampling to 2 Hz caused a large reduction in the accuracy of the RISE algorithm (62.5% ±1.4) but not the TSF algorithm (82.5% ±1.1).

When the algorithms trained on GCaMP6s normalised data were tested on CFA mice imaged with GCaMP8s, the prediction accuracy was somewhat reduced (RISE accuracy = 86.0% ±2.8, n = 2; Supplementary Fig. 4A, available at http://links.lww.com/PAIN/B956). Despite GCaMP8s being a more sensitive calcium indicator than GCaMP6s^40^ (Supplementary Fig. 4B, available at http://links.lww.com/PAIN/B956), this was caused by a reduction in sensitivity, ie, the ability of the algorithm to correctly identify spontaneously active neurons (70.3% ±4.4, n = 2).

Finally, the RISE algorithm trained on ΔF/F0 data predicted 31.5% (±4.8) of neurons to be active 1 day after CFA injection; n = 4 independent mice, which was significantly greater (*P* = 0.014; unpaired *t* test) than the number of spontaneously active neurons observed in control mice (10.2% ±1.1; n = 3; Fig. 2D-left). This was very similar to the number of neurons found to be spontaneously active by an experimenter, using the “gold standard” lidocaine method of identification (32.3% ±3.9; Fig. 2D—right).

### 3.3. Testing the robustness of the algorithm using additional data sets and disease models

**Figure 3.**
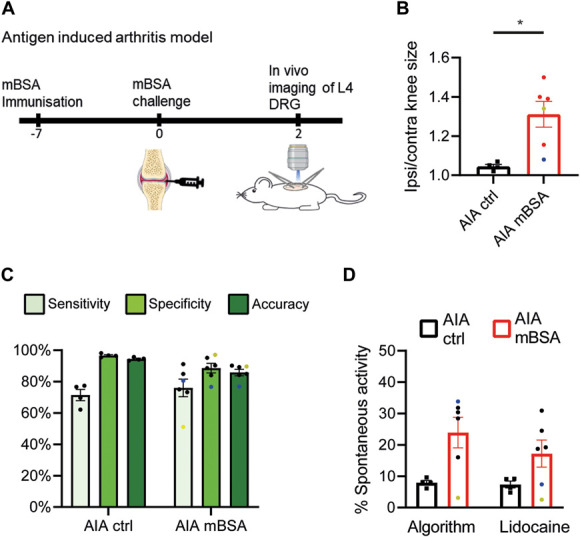
Assessment of spontaneous activity in an arthritis model. Experimental schematic illustrating how the AIA model is induced (A). The ratio of ipsilateral to contralateral knee size in mice 2 days after receiving an intraarticular injection of saline (ctrl) and mBSA (B). The results of the RISE algorithm's ability to detect spontaneous activity in the AIA model (C). The proportion of neurons in the L4 DRG with spontaneously activity in control and AIA animals on day 2 is shown in (D). RISE algorithm prediction is shown on the left, whereas conventional quantification by an experimenter using lidocaine-induced silencing is on the right. The yellow dots denote an experiment in which the GCaMP6s labelling efficiency was unusually low, ie, the laser power was double that of what is typical; consequently, the sensitivity was reduced in this experiment. The blue dots denote an experiment that had a bad movement artefact; consequently, the specificity was reduced in this experiment. n = 4-6/group ΔF/F0 normalised CFA data were used for training. Bar graphs represent mean ± SEM. Each dot represents data from one animal. * p<0.05.

Next, we tested the RISE algorithm's ability to predict when a neuron was firing spontaneously in an antigen-induced arthritis (AIA) model of joint pain (Fig. 3A). After immunisation, mice that underwent intra-articular injections of mBSA showed an increase in the size of the ipsilateral joint compared with its contralateral, uninjected counterpart (ipsi/contra ratio = 1.31 ±0.07; n = 6). This was significantly different (*P* = 0.011, unpaired *t* test) to the saline injected group (ipsi/contra ratio = 1.04 ±0.01; n = 4; Fig. 3B). Consistent with the results in the CFA model, the RISE algorithm accurately detected spontaneous activity in both treatment groups (accuracies = 85.9% ±2.1% and 94.4% ±0.5 in mBSA and control groups, respectively).

The yellow dot represents an experiment where the GCaMP6s labelling was suboptimal, ie, the laser strength required to visualise neurons was doubled compared with our standard settings (10% vs 5%). Although the knee was clearly inflamed in this animal (ispi/contral ratio = 1.33), very few neurons were spontaneously active (2.5% as determined by lidocaine), and hence, sensitivity of the RISE algorithm was reduced (50%). Nevertheless, thanks to its continuing high specificity, RISE appeared relatively robust to such a variation in labelling with overall accuracy levels only slightly lower than average (89.7%). By contrast, when specificity was reduced to 76.6%, due to a particularly bad movement artefact, the accuracy of the algorithm was more noticeably affected (accuracy = 76.9%; represented by blue dots in Fig. 3C).

Using the RISE algorithm, spontaneous activity was identified in 23.9% (±4.9; n = 6) of neurons following mBSA injection, which was similar to the percentage identified by an experimenter using lidocaine (17.2% ±4.3; Fig. 3D). There was a nonsignificant increase in the number of neurons identified with spontaneous activity in the mBSA group compared with the saline injected control group (7.9% ±0.7 active; n = 4; using RISE algorithm predictions, *P* = 0.29, unpaired *t* test; Fig. 3D). However, we were likely underpowered to detect any significant changes between the groups, due to our small sample size and the high variability in the proportion of spontaneously active neurons in mBSA mice.

**Figure 4.**
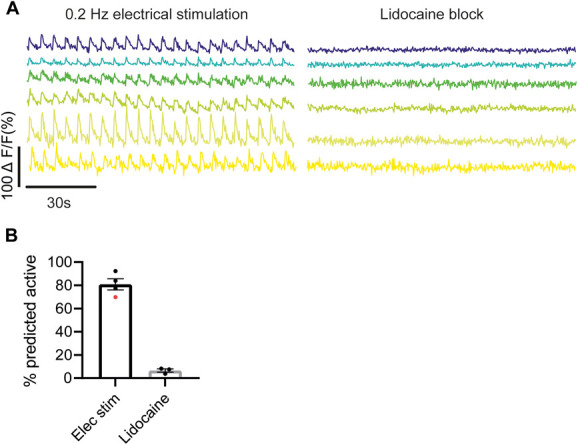
The RISE algorithm can detect the majority of neurons firing at low frequencies. Example ΔF/F0 normalised data traces of neurons firing at 0.2 Hz and after lidocaine block are shown in (A). Bar graph shows the proportion of neurons that were predicted active by the RISE classifier when stimulated at 0.05 to 0.2 Hz vs following lidocaine block (B). The algorithm could detect low-frequency neuronal activity, and very few neurons were predicted active when all neuronal activity was blocked by lidocaine. See also Supplementary Video 2. ΔF/F0 normalised CFA data were used for training. Bar graphs represent mean ± SEM. Each dot represents data from one animal. Red dot denotes experiment in which the nerve was electrically stimulated at 0.05 Hz; all other stimulations were performed at 0.2 Hz. CFA, complete Freund adjuvant; RISE, random interval spectral ensemble.

To determine whether the algorithm could detect low-frequency firing that was just above the level of background noise, the sciatic nerve was stimulated at suprathreshold strength at 0.05 to 0.2 Hz (Fig. 4A—left panel and see Supplementary Video 2). The RISE algorithm was able to detect low-frequency electrical activity 80.9% of the time (±4.8, n = 4; Fig. 4B). By contrast, very few neurons were predicted active when all activity was blocked by lidocaine (6.6% ±1.4, n = 4; Fig. 4C).

**Figure 5.**
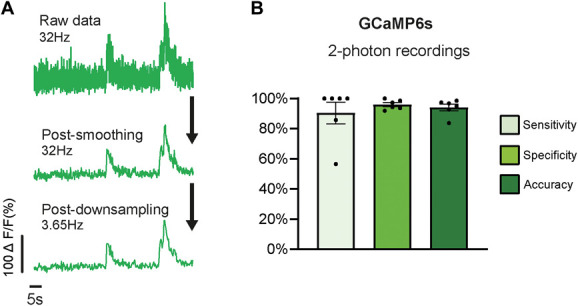
The RISE algorithm can detect GCaMP6s activity collected in a different laboratory using a different microscope configuration. Preprocessing steps necessary to convert 2-photon GCaMP6s data to a format that could be used for testing (A). Example of original ΔF/F0 normalised data traces (top) vs after smoothing (middle trace) and downsampling (bottom trace). The results of the RISE algorithm's ability to detect evoked activity in experiments performed with 2-photon microscope (B). Note that in this case, sensitivity, specificity, and accuracy were calculated using experimenter-determined response rates provided by the other laboratory in a blinded fashion (see methods for further details). Data collected from n = 6 mice. ΔF/F0 normalised CFA data were used for training. Bar graphs represent mean ± SEM. Each dot represents data from one animal. CFA, complete Freund adjuvant; RISE, random interval spectral ensemble.

To ensure that our algorithm was robust enough to detect neuronal activity in different experimental settings, we tested it on data collected in a different laboratory under different conditions, ie, both a different microscope configuration and acquisition rate. These 2-photon data required additional normalisation beforehand. We smoothed them using a moving average and downsampled the acquisition rate from 32 Hz to 3.65 Hz using a linear interpolation function in Python (Fig. 5A). The RISE algorithm could accurately detect activity in 1-minute-long 2-photon microscope recordings (accuracy = 94.0% ±2.2; 30-72 neurons/mouse, n = 6 mice; Fig. 5B), despite being trained on data recorded with a single photon microscope.

## 4. Discussion

Using traditional electrophysiological techniques to study spontaneous activity in sensory neurons has been challenging. In this study, we set out to determine whether in vivo calcium imaging can be used as an alternative means to study this type of activity in larger numbers of neurons at once. Indeed, we found that spontaneously active neurons can be visualised in 1-photon recordings using GCaMP indicators in a CFA model of pain. Using these data, we trained 2 supervised machine learning algorithms to identify whether either could successfully identify when a neuron was spontaneously active. We found that the RISE algorithm, trained on normalised ΔF/F0, performed particularly well. We tested it on independent CFA data and on an arthritis pain model and were able to demonstrate that it is robust enough for detecting inflammation-induced sensory neuron spontaneous activity in both cases. Furthermore, the algorithm can accurately predict activity on data collected by a different laboratory using a 2-photon microscope, suggesting that it is robust enough for detecting both spontaneous and evoked sensory neuron activity across different GCaMP6s data sets.

The proportion of neurons with spontaneous activity in control (∼10%) and CFA inflamed (∼32% on day 1) mice is broadly in line with that previously reported in the literature through electrophysiological studies in rats.^8,36,38^ So far, few studies have reported on spontaneous activity in sensory neurons using calcium indicators in vivo. In comparison to our baseline data, 2 studies using a GCaMP3 transgenic mouse line, reported much lower levels (0.13%-0.19%) of spontaneously active calcium transients,^12,17^ and another study using a GCaMP6s mouse line also found a lower proportion of neurons that were spontaneously active at baseline in anaesthetised mice (∼4%). These reports may have underestimated the true proportion of spontaneously active neurons because they used a standard deviation–based method for their analysis. We found this method to be less accurate for predicting spontaneous activity (Supplementary Fig. 1, available at http://links.lww.com/PAIN/B956). Another potential reason for why others reported lower levels of spontaneous activity could be because the overall acquisition rate for their recordings was slower in comparison to ours (<0.2-1.7 Hz vs 3.65 Hz). At the temporal resolution of 0.2 Hz, lower frequency firing could more easily be missed. Finally, differences in the levels of spontaneous activity could have been caused by differences in anaesthetic regimens: we used isoflurane in combination with urethane, whereas the study of Chen et al. used ketamine and xylene.^3^ Indeed, anaesthesia can impact peripheral neuron excitability, with the proportion of neurons with spontaneous activity reportedly higher (∼14%) in awake animals.^3^

There are many advantages of using in vivo imaging to study spontaneous activity over electrophysiology. One of the major challenges of using electrophysiology is that it is difficult to record spontaneously active nociceptive neurons (eg, compared with A fibres) because they are the smallest in size. By contrast, all sizes and subgroups of sensory neuron can be visualised using GCaMP calcium indicators,^5,34^ thereby reducing the bias toward recording from a particular fibre type. Electrophysiology is low throughput (tens of neurons per experiment), whereas we typically record from ∼400 to 500 neurons/DRG in an imaging experiment; therefore, imaging is not only faster but also the simultaneous recording of many neurons allows for the examination of coordinated patterns of spontaneous firing. Finally, neurons must be activated to visualise them when using electrophysiology, which can cause neuronal sensitisation and increase spontaneous firing when done repeatedly through receptive field testing.^1^ By contrast, no activation is required for visualising neurons before starting the recordings using in vivo imaging.

Besides its advantages, calcium imaging naturally also has limitations. GCaMP6 has relatively slow kinetics and therefore saturates quickly—around 0.5 Hz in DRG neurons in vivo.^5^ Therefore, it can be difficult to ascertain the number of action potentials fired during short higher-frequency bursts. This may be particularly important in the context of nociception because pain intensity ratings in humans have been reported to correlate with the frequency and duration of action potentials.^30^ Future work may be able to mitigate this limitation, eg, through machine learning–based algorithms that can predict the number of spikes produced from calcium transients for different indicators, eg, the CASCADE toolbox.^31^ Similarly, faster calcium indicators, such has GCAMP8^39^ or fast voltage indicators,^18^ may help improve our ability to resolve action potentials in imaging experiments going forward.

It is important to note that good signal to noise is required to visualise and detect low-frequency neuronal firing in in vivo imaging experiments with GCaMP6s. Reduced signal to noise can be caused by poor GCaMP labelling. This may result in a reduction in the number of spontaneously active neurons that can be visualised. Indeed, in one of our AIA experiments with particularly poor labelling intensity, only 2.5% of neurons were spontaneously active, despite the knee being clearly inflamed (knee size ratio = 1.33). Although the accuracy was not reduced in this experiment, the sensitivity was affected (see yellow dot in Fig. 3C), and this is likely because the prevalence of spontaneous activity (ie, the number of true positives) was so low that the number of false negatives has a greater impact on the outcome. Movement artefacts, eg, from breathing, can also introduce significant additional noise. Such artefacts can normally be mitigated by ensuring that the spinal vertebra are clamped adequately, so that the preparation is stable, and by using image registration algorithms. However, sometimes movement artefacts are difficult to completely remove, and this can lead to a reduction in the accuracy of detecting spontaneous activity using our trained machine learning algorithm (see example indicated by blue dots in Fig. 3). Therefore, we predict that our model maybe as, if not more, accurate at detecting activity in in vitro experiments because there are no movement artefacts, leading to improved signal to noise. By contrast, the model might be less accurate when in vivo imaging spinal cord neurons because the potential for movement artefacts is higher when imaging smaller ROIs at higher magnification.

Our trained model does have other limitations. We only used lidocaine to block activity and thus our algorithm only has been trained to detect spontaneous events that are dependent on voltage-gated sodium channels, ie, we may not be able to detect spontaneous events generated by calcium channels. We used the normalised ΔF/F0 data for training our algorithms because it produced accuracy results that were almost identical to that of the non-normalised data. One might find this somewhat surprising, given the baseline fluorescence that we are normalising against should already be raised in a spontaneously active neuron, thus decreasing the overall amplitude of any signal. However, this turned out not to be the case when examining inflammation-induced spontaneous activity because spontaneously active neurons fire at relatively low rates (<1 Hz) during inflammation,^8,26^ too low to cause saturation of GCaMP6s.^5^ Indeed, it seems unlikely that the algorithms trained on ΔF/F0 normalised data would be able to accurately predict spontaneously active neurons firing at higher frequencies (>5 Hz), which is a caveat of using our currently trained versions. However, because there are now faster and more sensitive GCaMPs, which are reported to be able to follow firing frequencies of ≥10 Hz,^40^ it seems likely that this obstacle can be overcome by retraining the algorithms on novel data collected using these indicators.

We will continue to improve the robustness of our algorithm by retraining with data generated with different indicators. This will be necessary because we have found that with its current training set based on GCaMP6s data, the RISE algorithm does not generalise well in its ability to detect activity when it is derived from neurons containing GCaMP8s as an indicator. Machine learning algorithms are limited to understanding only the data they are trained on. The pretrained RISE algorithm documented here is therefore specific to detecting GCaMP6s calcium transients. Algorithmic detection of spontaneous activity using indicators that have different kinetics to GCaMP6s, such as GCaMP8s,^40^ will require a model retraining step with ground truth data generated using the same indicator used to generate the test data.

To make our algorithm easy for others to use, we have generated a cloud-based version that is available as a Google Colaboratory Notebook. It is recommended that input data are acquired between 3 and 32 Hz and is as close to 5 minutes in duration as possible (but not longer). Both spontaneous and evoked GCaMP6s activity should be detectable with our current algorithm. Users can also use the Notebook to retrain the algorithm based on their own training sets—however, this will require them generating their own “ground-truth” activity data, for example, using lidocaine.

## 5. Conclusion

We conclude that in vivo imaging with GCaMP indicators is a suitable technique for detecting spontaneous activity in models of pain. We have overcome the difficulty of differentiating spontaneous activity from noise using a machine learning algorithm, which, when trained, can accurately detect GCaMP6s activity in both 1-photon and 2-photon recordings. Our trained algorithm will provide a useful tool for those performing in vivo imaging experiments in the pain and wider-neuroscience community.

## Conflict of interest statement

The authors declare no conflicts of interest in relation to this work.

## Appendix A. Supplemental digital content

Supplemental digital content associated with this article can be found online at http://links.lww.com/PAIN/B956. Supplemental videos can be found on the *PAIN* Web site.

## Supplementary Material
